# An Evolutionary-Network Model Reveals Stratified Interactions in the V3 Loop of the HIV-1 Envelope

**DOI:** 10.1371/journal.pcbi.0030231

**Published:** 2007-11-23

**Authors:** Art F. Y Poon, Fraser I Lewis, Sergei L. Kosakovsky Pond, Simon D. W Frost

**Affiliations:** 1 Department of Pathology, University of California San Diego, La Jolla, California, United States of America; 2 Institute of Evolutionary Biology, University of Edinburgh, Edinburgh, Scotland, United Kingdom; Harvard University, United States of America

## Abstract

The third variable loop (V3) of the human immunodeficiency virus type 1 (HIV-1) envelope is a principal determinant of antibody neutralization and progression to AIDS. Although it is undoubtedly an important target for vaccine research, extensive genetic variation in V3 remains an obstacle to the development of an effective vaccine. Comparative methods that exploit the abundance of sequence data can detect interactions between residues of rapidly evolving proteins such as the HIV-1 envelope, revealing biological constraints on their variability. However, previous studies have relied implicitly on two biologically unrealistic assumptions: (1) that founder effects in the evolutionary history of the sequences can be ignored, and; (2) that statistical associations between residues occur exclusively in pairs. We show that comparative methods that neglect the evolutionary history of extant sequences are susceptible to a high rate of false positives (20%–40%). Therefore, we propose a new method to detect interactions that relaxes both of these assumptions. First, we reconstruct the evolutionary history of extant sequences by maximum likelihood, shifting focus from extant sequence variation to the underlying substitution events. Second, we analyze the joint distribution of substitution events among positions in the sequence as a Bayesian graphical model, in which each branch in the phylogeny is a unit of observation. We perform extensive validation of our models using both simulations and a control case of known interactions in HIV-1 protease, and apply this method to detect interactions within V3 from a sample of 1,154 HIV-1 envelope sequences. Our method greatly reduces the number of false positives due to founder effects, while capturing several higher-order interactions among V3 residues. By mapping these interactions to a structural model of the V3 loop, we find that the loop is stratified into distinct evolutionary clusters. We extend our model to detect interactions between the V3 and C4 domains of the HIV-1 envelope, and account for the uncertainty in mapping substitutions to the tree with a parametric bootstrap.

## Author Summary

The third variable loop (V3) of the human immunodeficiency virus type 1 (HIV-1) envelope is a principal determinant of viral growth characteristics and an important target for the immune system. Interactions between residues of V3 allow the virus to shift between combinations of residues to escape the immune system while retaining its structure and functions. Comparative study of HIV-1 V3 sequences can detect such interactions by the covariation of sites in the sequence, which can then be used to inform vaccine development, but current methods for detecting such associations rely on biologically unrealistic assumptions. We demonstrate that these assumptions cause an excessive number of spurious associations, and present a new approach that couples phylogenetic and Bayesian network models, and greatly reduces this number while retaining the ability to detect real associations. Our analysis reveals that the V3 loop is stratified into discrete layers of interacting residues, suggesting a partition of functions along this viral structure with implications for vaccine development.

## Introduction

The human immunodeficiency virus type 1 (HIV-1) possesses a highly variable envelope comprising the glycoproteins gp120 and gp41, which mediate the binding and entry of the virus into a host cell. The viral envelope is also a potent antigen for neutralizing antibodies [1–4] and cytotoxic and helper T lymphocytes [5–7], which is manifested as extensive sequence divergence in the *env* gene [8,9]. Consequently, HIV-1 is required to maintain a functioning envelope while accumulating a sufficient number of mutations in *env* to escape the adaptive immune response. This conflict can be surmounted by the evolving virus populations through selection for combinations of substitutions that exploit structural or functional interactions among residues in the envelope glycoproteins [10]. A structural interaction occurs between residues that cooperate in the formation and stabilization of secondary or tertiary protein structures. On the other hand, a functional interaction is a statistical association that arises indirectly between residues that participate in the same protein function, e.g., key residues in a conformational binding site or glycosylation motif. Redundancy that arises from such interactions allows residues to be replaced by other combinations while conserving the overall phenotype. This phenomenon, known as compensatory mutation, features prominently in HIV-1 evolution [11–13] and is pervasive across all levels of biological diversity [14].

The detection of interactions among residues in rapidly evolving viral proteins such as the HIV-1 envelope is an important and unresolved problem. First of all, the failure to account for such interactions can hamper efforts to map genetic variation to virus phenotypes, such as coreceptor usage, neutralization sensitivity, or drug resistance. For example, a substitution at position 306 in HIV-1 gp120 (relative to the HXB2 reference sequence) is necessary, but not sufficient, to induce a shift in coreceptor usage in HIV-1; full expression of this phenotype requires additional substitutions at positions 320 or 324 [15,16]. Second, the identification of interacting residues may be applied toward defining a minimal set of HIV-1 protein sequences to be incorporated into a broadly reactive vaccine [17,18]. Consequently, a substantial literature has developed around the goal of defining an accurate map of interactions in the HIV-1 envelope [19–22]. The majority of these studies have focused on detecting interactions within the third variable domain of the external envelope glycoprotein gp120.

The third variable domain (V3) of the HIV-1 envelope typically spans 33 to 35 residues that are bounded by two invariant cysteines that form a disulfide bond to create a loop. The V3 loop is characterized by extensive sequence variation, and is a principal determinant of important HIV-1 phenotypes such as coreceptor usage and cell tropism [23–25]. Neutralizing antibodies elicited by the HIV-1 envelope are often directed against epitopes in the V3 loop [1,3], and exposure to synthetic V3 peptides is sufficient to raise strain-specific neutralizing antibodies against lab-adapted strains of HIV-1 [2]. On the other hand, broadly reactive and potent neutralizing antibodies tend to recognize conformational rather than linear epitopes on V3 [26]. Because of its functional and antigenic importance, the three-dimensional structure of V3 has been studied extensively [27,28], revealing a flexible, solvent-accessible loop that protrudes outward from the gp120 core toward the host cell.

To date, comparative studies of HIV-1 *env* V3 have looked for evidence of residue–residue interactions by measuring covariation among positions in a sample of nucleotide (i.e., codon) or protein sequences [19–21,29]. Sequence covariation is most frequently assayed by the application of one or more pairwise association test statistics, e.g., mutual information [19]. The resulting set of statistically significant pairwise associations is conventionally adjusted for multiple comparisons, either using the conservative Bonferroni correction [20] or the Benjamini-Hochberg false discovery rate (FDR) method [21,30]. This procedure is straightforward and yields a set of putative interactions, but implicitly requires a number of unreasonable assumptions. First, by treating each sequence as an independent observation in a random sample, the procedure ignores their evolutionary history. However, it is well known that shared ancestry will produce spurious correlations between jointly inherited character states [31,32]. This phenomenon has more recently been found to substantially alter the results of a landmark study into genetic associations of HIV-1 escape from cytotoxic T lymphocytes in a human population [33,34]. Secondly, the pairwise associations that are selected by the test statistic have never been evaluated in the context of any other residue. For example, an interaction between two residues may be dependent on the residue at a third position in the V3 loop. Many of the test statistics employed by previous studies are inherently unable to model such higher-order associations, requiring that we assume such interactions do not exist. Because each association is evaluated in complete isolation, we are left with a “laundry list” of pairwise associations with no apparent procedure for compiling these into a meaningful overall picture of interactions in the V3 loop.

In this study, we propose a new method for detecting interactions from an arbitrary sample of genetic sequences that relaxes both of these assumptions. We apply our method to analyzing residue–residue interactions in the V3 loop of HIV-1 gp120, which has emerged as a model system for the implementation of association test statistics or classification algorithms [16,19,20,29,35]. Instead of quantifying covariation in the observed set of sequences *D*, we will take their evolutionary histories as our data [36–38]. Because these data are almost always unobservable, they must be inferred from *D* by assuming that the sequences have evolved according to some stochastic model *M*. We will also assume that the phylogenetic tree *T*, which defines the common ancestry of extant sequences, is known. Using maximum likelihood, we can infer the ancestral sequences *D*′ at each branching point, or node, of the tree *T* as a function of *D* and *M* [39,40]. Any difference between sequences in *D* ∪ *D′* occupying adjacent nodes of *T* implies that one or more substitution events occurred at that site in the intervening branch [41]. The end result is a sample of evolutionary events encoded as a matrix *D*′′, in which each unit of observation (row) corresponds to a branch in the phylogenetic tree onto which substitutions are mapped. Each column of *D*′′ corresponds to a unique codon position, containing a “1” for every branch in which one or more substitutions occur at that position, and “0” otherwise. In sum, *D′′* is a phylogenetically independent sample of nonsynonymous substitution events that is derived by augmenting the observed data *D* with an evolutionary model and a tree.

To address the second assumption, we will analyze the phylogenetically augmented data *D′′* as a Bayesian network. A Bayesian network, B, is a graph that encodes a set of conditional independence assumptions on the joint probability distribution of random variables [42]. A graph is a visual depiction of relationships between unique objects that typically assumes the form of points (nodes) connected by line segments or arrows (edges). In this case, each node represents a random variable whose outcome may depend on other variables. For example, a directed edge originating from node *A* and terminating at node *B* (*A* → *B*) represents the probabilistic assumption *P*(*A* ∩ *B*) = *P*(*A*) *P*(*B* | *A*) ≠ *P*(*A*) *P*(*B*), i.e., that *B* is conditionally dependent on *A*. The set of all edges in the graph corresponds to the “structure” of the Bayesian network, B*_S_*. Bayesian networks hold a considerable advantage over pairwise association tests. First of all, pairwise association tests are unable to distinguish between direct and indirect associations between variables. For instance, consider the case in which two nodes are each dependent on a third (*B* ← *A → C*). Association test statistics are susceptible to attributing significant associations to all three pairs. However, *B* is conditionally independent of *C*, given *A*; i.e., *P*(*B* ∩ *C* | *A*) = *P*(*B* | *A*) *P*(*C* | *A*). Because conditional independence is explicitly encoded by a Bayesian network, we can obtain a more parsimonious and informative representation of biological causation [43]. Secondly, Bayesian networks provide an efficient representation of the joint distribution in an accessible graphical format. It is therefore not necessary to assemble an ad hoc summary network from a list of significant pairwise associations.

We apply this “evolutionary-network” model to detect interactions among residues in the V3 loop of the HIV-1 envelope. Using maximum likelihood, we infer a phylogenetically independent set of substitution events. Interactions among residues are manifested as correlated substitutions within this inferred set, such that substitutions affecting a subset of residues tend to be mapped to the same branch of the tree. Because the phylogenetic inference of substitution events is susceptible to some uncertainty, we carry out a parametric bootstrap procedure to quantify the sensitivity of the results from a maximum-likelihood reconstruction. We also apply our method to several control cases, including the better-characterized compensatory interactions in HIV-1 protease, to validate our results for the V3 loop. Our analysis reveals a large number of interactions among residues that fall into stratified clusters along the length of the V3 loop.

## Materials and Methods

### Data

A total of 1,154 full-length sequences of HIV-1 *env* were obtained from the Los Alamos National Laboratory (LANL) HIV sequence database (http://www.hiv.lanl.gov), excluding recombinant sequences and limited to one sequence per patient. The nucleotide alignment was adjusted using Se-Al (Andrew Rambaut, http://tree.bio.ed.ac.uk/software/seal). According to the LANL subtype annotation, this alignment comprised 500 (43.3%) subtype C, 431 (37.3%) subtype B, 109 (9.4%) subtype A, 65 (5.6%) subtype D, 25 (2.2%) subtype G, 17 (1.5%) subtype F, three (0.3%) subtype H, two (0.2%) subtype K, and two (0.2%) subtype J sequences. Using the “11/25 rule” [16,44], we predicted that 131 sequences encoded V3 loops binding the CXCR4 coreceptor; this subset comprised predominantly subtypes B and D (*n* = 105). We used the nucleotide alignment to reconstruct a phylogeny by neighbor-joining [45] using Tamura-Nei distances [46] with rate variation across sites (parameterized by a gamma distribution with a shape parameter α = 0.5), excluding the indel-rich variable domains of gp120 to avoid the confounding effects of uncertainty in alignment. We removed nine sequences that were identical in the portion of the alignment applied toward reconstructing the phylogeny.

### Mapping Substitutions to the Tree

We fit a codon substitution model [47] combined with the general time-reversible nucleotide substitution model (GTR) [48] to the alignment and tree by maximum likelihood using HyPhy [49]. The branch lengths of the nucleotide tree were constrained to be scaled by a constant factor, reducing considerably the number of parameters to be estimated; this has been previously demonstrated to be a robust approximation for fitting codon models [50,51]. Maximum-likelihood reconstructions of ancestral sequences were extracted from the fitted model for each internal node of the tree. We inferred that one or more nonsynonymous substitutions had occurred along a branch if the reconstructed codon states at the nodes at either end of the branch encoded different amino acids [41,51]. Each branch of the tree was thereby annotated with a binary-state vector, according to the presence or absence of a nonsynonymous substitution in that branch. This procedure yielded a matrix comprising 2,305 rows and 33 columns. To quantify the uncertainty in the reconstruction of ancestral sequences, we generated parametric bootstraps in HyPhy by resampling ancestral sequences in proportion to their likelihood [41,51] given the parameter estimates of the evolutionary model (i.e., branch lengths and codon substitution rates), resulting in 100 replicate matrices that were analyzed alongside the original matrix using Bayesian networks, as described in the next section.

### Bayesian Networks

We analyzed the matrix of substitution events mapped to branches in the phylogeny (*D*′′) as a Bayesian network comprising 33 discrete nodes, using an algorithm proposed by Friedman and Koller [52] that we implemented in HyPhy. The problem of detecting interactions among codon positions is equivalent to “learning” the structure B*_S_* of a Bayesian network B from data *D′′* [53], in which the structure refers to a set of directed edges representing conditional dependencies between nodes. According to Robinson's [54] recursive formula, there are approximately 2.67 × 10^190^ possible network structures on 33 nodes; this number clearly precludes an exhaustive search for an optimal structure. Furthermore, more than one network structure may be supported equivocally by the data, especially when the number of observations is small relative to the number of nodes [52]. Friedman and Koller proposed carrying out a Markov Chain Monte Carlo (MCMC) algorithm over the space of node orders rather than network structures. A node order is a permutation of the nodes in a linear sequence such that a node can only become assigned as a “parent” of nodes that are positioned to its left (i.e., “precedes,” ≺). For example, the node order *A* ≺ *B* defines a subset of network structures that excludes all structures containing the edge *A* ↮ *B*. The node order space is relatively more compact—for instance, there are approximately 8.6 × 10^36^ permutations of 33 nodes—and yields a smoother posterior probability surface with improved MCMC convergence properties [52].

Following Friedman and Koller [52], our implementation precomputed the posterior probability, or score, for every combination of states assigned to the parental nodes of the *i*th node, for all *i*. These node scores were cached into memory and accessed by direct indexing to make subsequent calculations more efficient. The posterior probability of a structure B*_S_* was calculated according to the K2 scoring metric [55], which integrated over the conditional probabilities at each node (i.e., the network parameters, B*_P_*) that were distributed according to an uninformative Dirichlet prior, i.e., a uniform distribution over the interval [0, 1] for binary-valued nodes. The K2 metric tends to favor more parsimonious, and hence more interpretable, network structures than alternative scoring metrics such as the Bayesian Dirichlet metric or BDe [56]. (The BDe metric yielded nearly identical results to the K2 metric when the “imaginary” prior sample size required by the BDe was set to near zero.) Our use of cached node scores exploited the fact that the posterior probability of a network structure decomposes into a product of node “families,” which blocks the peripheral structure by accounting for the direct influence of the parents of each node. We ran a single Markov chain initialized with a random permutation of the node ordering. At each step, a proposal function swapped two random nodes in the current node order. The proposed node order was accepted unconditionally if its posterior probability exceeded that of the current ordering, or conditionally in proportion to the ratio of posterior probabilities (i.e., Metropolis-Hastings sampling [57]).

We ran the Markov chain for 10^6^ steps with a burn-in period of 10^5^ steps, which we have found to be more than sufficient for convergence for networks of this size. We ran a duplicate Markov chain and found that Gelman and Rubin's convergence diagnostic [58] was indistinguishable from one, which was consistent with convergence. Node order statistics (e.g., edge posterior probabilities) were sampled at 10^4^ steps of the chain at equal intervals. We estimated the posterior probability for each of 528 possible edges as the proportion of structures in the sample in which the edge was present in either direction, weighted by *P*(B*_S_* | *D*) [52]. A consensus network structure was assembled from all undirected edges with a marginal posterior probability exceeding the cutoff value 0.95. The same analysis was carried out for each parametric bootstrap sample, except the chain in each case was run for 10^5^ steps with a burn-in period of 10^4^ steps and 10^3^ samples. The profile of each chain was visually inspected to evaluate convergence. The frequency of edges with a posterior probability exceeding 0.95 was summed across bootstrap samples to quantify the sensitivity of edges in the maximum-likelihood consensus network to uncertainty in the reconstruction of ancestral sequences.

### Model Validation

We employed three validation procedures to evaluate the accuracy of our methods. First, we invoked a paired binary-character model, originally developed to analyze the evolution of N-linked glycosylation site motifs [22], in order to simulate the evolution of V3 sequences along the “observed” phylogeny with a known set of interactions. This model specifies the substitution rates between the paired states {00, 01, 10, 11}, disallowing simultaneous substitutions affecting both sites in a pair, i.e., 00 <≠> 11, 01 <≠> 10. We converted our alignment of V3 sequences into binary-character sequences based on the presence or absence of the alignment consensus amino acid at each position of the sequence. For example, any V3 sequence that encoded a threonine at position 1 was converted into a binary string beginning with “1,” and “0” otherwise. We constrained the substitution rate parameters of the paired binary-character model such that the expected frequency of “1”s in simulated alignments would equal the observed frequency of consensus residues in our V3 alignment. A “pairwise coevolution” parameter, ɛ, determined the factor by which a “1” at one site accelerated the rate of “0” → “1” substitutions at the second site, or conversely the odds that a “0” at one site became replaced by a “1” with respect to the presence or absence of a “1” at the second site of a pair. In other words, if ɛ = 1, then the paired model was effectively equivalent to a model in which every site evolved independently. We simulated the evolution of binary sequences comprising 17 coevolving pairs of sites (i.e., {1, 2}, {3, 4},..., {33, 34}) along the original neighbor-joining tree. Two hundred replicate alignments were generated in this fashion for a range of ɛ parameter values. Each alignment was analyzed by the evolutionary-network method outlined above, i.e., by fitting a model of independently evolving binary characters by maximum likelihood, assigning substitution events to branches in the tree, and analyzing the distribution of substitutions as a Bayesian network using an MCMC analysis. We recorded the frequency that edges in the network with marginal posterior probabilities exceeding 0.95 recovered our predefined paired interactions (true positives) or spurious ones (false positives). These results were contrasted with the rates of true and false positives obtained from using the Fisher exact test on the simulated binary sequence alignments (i.e., without correcting for the phylogeny). We chose the Fisher exact test as a representative example of pairwise association test statistics.

Second, we simulated the evolution of nucleotide sequences along the tree according to a more realistic codon substitution model whose parameters were estimated from the original alignment of V3 sequences. We randomly generated 100 replicate alignments with the same dimensions and characteristics (e.g., expected codon frequencies) as our observed V3 alignment by this method. Because the codon substitution model assumes that an alignment is a set of independently evolving codon sites [47], any significant interactions between sites were false positives caused by founder effects in the phylogeny. We evaluated the false-positive rate for our method against the rate for a more conventional pairwise association test, in which we applied the Fisher exact test to every pairwise combination of codon positions in the simulated V3 loop sequences, enumerating consensus and nonconsensus residues to generate a 2 × 2 contingency table.

Third, we applied the evolutionary-network model to a set of HIV-1 protease sequences, in which compensatory interactions are substantially better characterized empirically or structurally than the V3 loop, particularly in the context of drug resistance [11,12,59–64]. We obtained an alignment of 2,641 HIV-1 subtype B sequences from the Stanford HIV resistance database (http://hivdb.stanford.edu) [65] representing patients on active drug regimens that included at least one protease inhibitor. This alignment was analyzed using the evolutionary-network methods, and the results were contrasted with known interactions from the empirical and structural literature.

### Structural Visualization

We mapped interactions from the consensus Bayesian network to a three-dimensional structure of the V3 loop of HIV-1 gp120 complexed to the CD4 receptor and X5 antibody (Research Collaboratory for Structural Bioinformatics Protein Data Bank [RCSB PDB]), using the visualization software Chimera (University of California San Francisco, Computer Graphics Lab [66]).

## Results

### Reconstruction of Nonsynonymous Substitutions

The maximum-likelihood reconstruction of ancestral sequences along the tree resulted in approximately 1.87 nonsynonymous substitutions per branch. The mean number of inferred nonsynonymous substitutions was significantly divergent between internal (0.48 substitutions per branch) and terminal (3.28) branches of the tree (Wilcoxon rank sum test, *W* = 152041, *p* ≪ 0.001); only 95 out of 1,142 (8.3%) internal branches had more than one substitution mapped. We found substantial variation in the total number of nonsynonymous substitutions among codon positions in V3 (coefficient of variation, C.V. = 1.16). The largest number of inferred substitutions occurred at residue 24, whereas residues 2, 16, 27, and 32 were highly conserved (numbered according to their position within the interval bounded by cysteines in the consensus sequence, as in [19]). This distribution was consistent with the pattern of diversifying selection across sites (unpublished data), implying that the differences were not simply due to variation among codon positions in mutation rate or the expected number of nonsynonymous sites. Adjusting the inferred number of nonsynonymous substitutions for the expected number of nonsynonymous sites at each codon position, and normalizing by the analogous quantity for synonymous substitutions (i.e., *dN* − *dS*), indicated that residues 13 and 29 were even more strongly conserved than implied by the uncorrected frequency of nonsynonymous substitutions. The detection of interactions among codon positions in the number of nonsynonymous substitutions did not require any such correction, however.

### Model Controls

**Figure 1:**
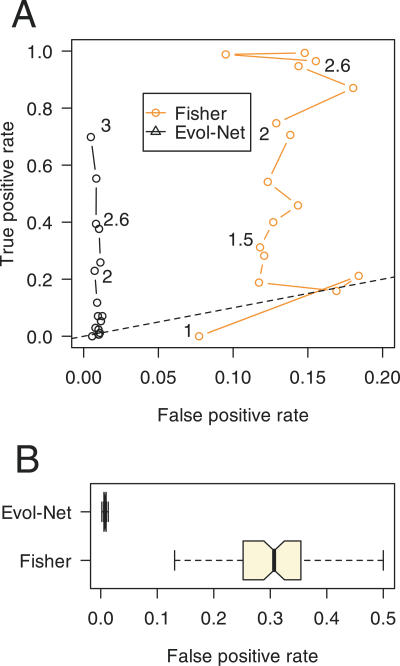
True- and False-Positive Rates on Simulated Data (A) A receiver operating characteristic (ROC)-like curve, in which the *y*-axis corresponds to the true-positive rate (TPR), and the *x*-axis corresponds to the false-positive rate (FPR). A perfect analytical method would be located in the upper-left corner (i.e., TPR = 1, FPR = 0), whereas methods located near the diagonal (dashed line, TPR = FPR) are statistically equivalent to a random guess. Each point in the plot corresponds to the mean outcome from the corresponding model analysis (orange = the Fisher exact test, black = evolutionary-network) of ten replicate simulations of binary-state sequences evolving under various settings of the pairwise coevolution parameter, ɛ (ranging from 1 to 3; see figure labels). (B) Boxplots corresponding to the false-positive rate (as a fraction of the total number of pairwise comparisons = 528) from the corresponding analysis (evolutionary-network [Evol-Net] or Fisher exact test [Fisher]) of simulated sequences evolving according to a null model of codon substitutions in which sites evolve independently, using parameter settings estimated from the original V3 sequence alignment. We generated 100 replicate simulations of nucleotide sequences evolving along the original neighbor-joining tree.

We validated the accuracy of our evolutionary-network model using three different controls. First, we simulated the evolution of HIV-1 V3-like sequences along the original phylogeny as vectors of binary characters switching between consensus and nonconsensus residues. Each consecutive pair of residues was constrained to coevolve according to an adjustable parameter ɛ, where ɛ = 1 corresponded to independently evolving sites. We contrasted the performance of a binary-state analog of the evolutionary-network model, reconstructing substitution events by maximum likelihood, against the results from applying the Fisher exact test to the extant binary sequences (Figure 1A). After correcting for multiple comparisons using the Benjamini-Hochberg correction [30], we found that the Fisher exact test resulted in a very high number of false positives increasing with the pairwise interaction parameter ɛ (with means ranging from 42 to 100 false positives out of 544 negative instances over the range of ɛ from 1 to 3). Although the Fisher exact test appeared to recover more true positives on average than our evolutionary-network model for a given value of ɛ, our model sustained a substantially lower rate of false positives (averaging five out of 544 negative instances). As a result, our model converged to the most desirable outcome (100% true-positive rate and 0% false-positive rate) with increasing values of ɛ, whereas the Fisher exact test diverged closer to the line of no discrimination (i.e., random guess; Figure 1A). Similar results were obtained using a more conservative Bonferroni correction for the Fisher exact tests.

Second, we simulated the evolution of HIV-1 V3 sequences along the phylogeny using a more realistic codon-based substitution model. Because this model assumes that each codon site evolves independently, the number of significant associations from each replicate simulation provided an estimate of the false-positive rate. Using the Fisher exact test on the pairwise combination of amino acids in simulated sequences, we found false-positive associations between 160.8 out of 528 pairs on average (10% and 90% quantiles = 115.4 and 217.3, respectively; Figure 1B). This number of significant pairwise associations corresponded to an expected false-positive rate of about 30.4% (10% and 90% quantiles = 21.8% and 41.2%, respectively), predominantly due to the common ancestry of sequences (i.e., founder effect [34]). In contrast, our evolutionary-network analysis yielded about four false positives on average (0.8%; Figure 1B), corresponding to a 40-fold improvement in specificity.

Third, we applied our evolutionary-network method to analyze HIV-1 subtype B protease sequences isolated from 2,461 patients undergoing drug regimens including at least one protease inhibitor [65]. HIV-1 protease is better characterized structurally than the relatively flexible V3 loop of the envelope glycoprotein gp120 [12,63,67]. In addition, compensatory interactions between several sites in HIV-1 protease are extensively documented from clinical and experimental studies [11,12,59–64], with greater consistency among studies than interactions in V3. We obtained a consensus network comprising 16 edges with marginal posterior probabilities exceeding a cutoff of 0.95 (Figure S1). Two nodes representing the codon sites M46 and V82—prefixed with the alignment consensus residue and numbered according to their position in the HXB2 reference sequence—were highly connected by five (L10, V32, T74, V82, L90) and four (M46, V48, I54, A71) edges, respectively. Both M46 and V82 are well-known sites of mutations that interact with mutations at the other sites identified in this network in order to confer resistance to protease inhibitors and cross-resistance to multiple inhibitors [11,12,62,63]. We also recovered a highly significant edge between the codon sites D30 and N88, at which mutations have been jointly implicated in clinical data as conferring specific resistance to the inhibitor nelfinavir [68]. In sum, we found strong concordance between our network and clinical and experimental studies of compensatory mutations in HIV-1 protease.

### The Consensus Network Stratifies V3

**Figure 2:**
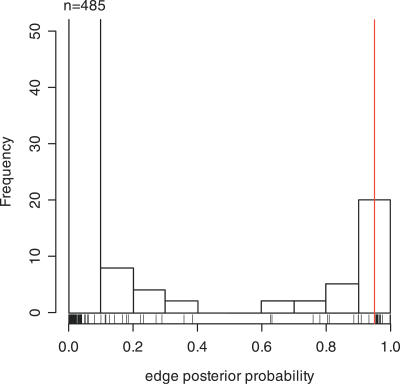
Distribution of the Marginal Posterior Probability of Network Edges This histogram indicates the frequency of the marginal posterior probability of the 528 possible edges, sampled from a Markov chain over the space of node orders (see Materials and Methods). The prior probability of each edge was set to the uninformative value of 0.5. Note that the vertical range of the histogram was truncated to a maximum of 50; the first bin of the histogram contained 485 edges as indicated on the figure. Individual values are indicated by tick marks along the *x*-axis. A red line indicates the arbitrary cutoff value of 0.95.

**Figure 3:**
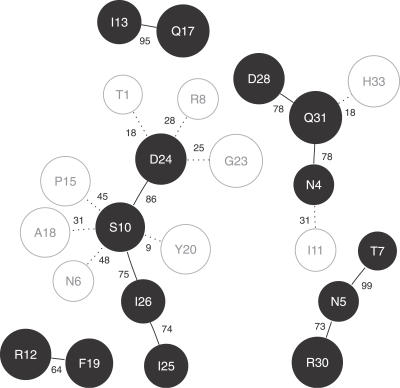
The Consensus Network of V3 Residues Each node corresponds to a residue in the V3 loop, numbered according to their position in the consensus sequence (identical to Korber et al. [19]) and labeled with the consensus amino acid. Dark-shaded nodes indicate residues that are connected by edges with parametric bootstrap support values exceeding 50%; each edge is labeled with its support value. Edges with support values below this threshold are indicated by dashed lines.

The prior probability of every potential edge was set to 0.5. Given our augmented dataset, the distribution of the posterior probabilities of edges was strongly U-shaped, with a distinct cluster of edges with probabilities exceeding 0.95 (Figure 2). This outcome indicated that our phylogenetically augmented data matrix *D*′′ was sufficiently informative to distinguish network edges supported by the data from edges with little support. The consensus network assembled from edges above our cutoff comprised five components, including one large network component connecting 11 nodes (Figure 3). All putative interactions identified by the consensus network were “positive” (odds ratio [OR] > 1), such that a substitution at one residue significantly increased the probability of a substitution at a different residue in V3. We caution that because nonsynonymous substitutions on branches were relatively rare events, our analysis may have been subject to an intrinsic lack of power to detect negative interactions, i.e., where the occurrence of one substitution excluded substitutions at other sites. The parametric bootstrap support values for each edge in the consensus network are provided in Figure 3 as integer values (between 0 and 100) adjacent to each edge. These values quantified the sensitivity of each edge to uncertainty in the reconstruction of ancestral sequences. Nine of the edges in the network had support values below a cutoff of 50; these edges were trimmed from the final consensus network.

**Figure 4:**
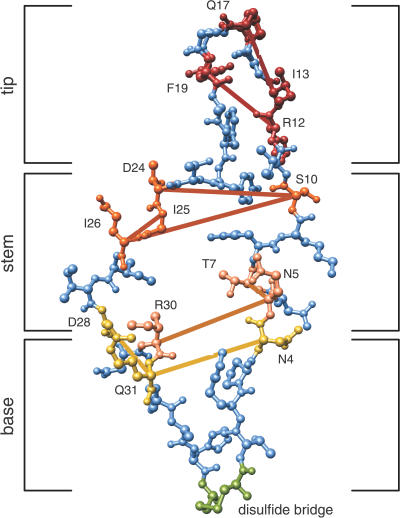
Structural Model of the V3 Loop A three-dimensional visualization of the structure of the V3 loop, using structural coordinates from a model comprised of the HIV-1 gp120 core protein complexed to the CD4 receptor and the X5 antibody [28]. The structure is oriented such that the host cell membrane would be positioned at the top of the figure. The cysteine residues forming a disulfide bridge that closes the loop are labeled in green. The amino acid sequence depicted here differs from our consensus sequence at five positions, such that identity would require the following substitutions: Q5N, H12R, R17Q, T21A, and E24D.

The strongest association in the consensus network occurred between residues 5 and 7 (OR = 155.6), which jointly defined a conserved N-linked glycosylation site motif (i.e., NNTR). Upon inspection, we found 28 phylogenetically independent events in which substitutions occurred along the branch, affecting both residues and disrupting the motif. Because a substitution at either residue would have been sufficient to eliminate the N-linked glycosylation site motif, this association suggested the presence of additional constraints on V3 in the absence of glycosylation. We also found evidence of an interaction between residues 5 and 30 (OR = 53.4). Although these residues resided on the opposite strands of the V3 loop, they were roughly equidistant from the base (Figure 4), which may facilitate an interaction bridging the loop.

Two of the network components (R12–F19 and I13–Q17) represented positive associations that were nested with respect to the secondary structure (Figure 4), which was consistent with stratification of the V3 loop. Both putative interactions were located in the region identified as the “tip,” which has been implicated in initiating gp41-mediated fusion with the host cell membrane, in addition to acting as the binding site for several monoclonal antibodies [28].

A large component comprised associations among the nodes S10, D24, I25, and I26, which all mapped to residues in the stem region of the V3 loop [28]. This component included a strongly supported association (in 86 out of 100 parametric bootstrap samples) between residues 10 and 24, which has been detected in previous studies of covariation in V3 loop sequences [19,20]. These residues have been implicated as strong determinants of coreceptor usage, i.e., the 11/25 rule [44], and act synergistically to alter the syncytium-induction phenotype [15]. Associations between residue 24 and the adjacent residues 25 and 26 were blocked by residue 10 (Figure 3). Indeed, when we repeated the analysis with a ban on edges between the nodes S10 and D24, node D24 nonetheless failed to become incorporated into the same component as I25 and I26. Overall, putative interactions between adjacent residues were more the exception than the rule; the majority of the putative interactions tended to bridge opposite strands of the V3 loop.

We applied the 11/25 rule to classify 131 of the extant sequences as yielding CXCR4-binding virus, i.e., having an “X4” phenotype. Thirty-seven of the X4 sequences formed monophyletic groups, for which each common ancestor may have been interpreted to be X4 also. On the contrary, each X4 sequence would most likely have been derived from a CCR5-binding ancestor over the course of an infection [25]. Parsimonious models of evolution would consequently have been susceptible to underestimating the number of substitutions at positions 10 and 24. Indeed, the maximum-likelihood reconstruction at groups comprising X4 sequences predicted that the majority of ancestors were X4 also; we found only two cases of an X4 motif being evolved independently in sequences derived from a CCR5 ancestor. Despite this outcome, our reconstruction also assigned substitutions at positions 10 or 24 to nearly half (15 of 33) of the terminal branches with an X4 ancestor. Because even one basic residue at either 10 or 24 is sufficient to fulfill the 11/25 rule, many substitutions replaced a redundant residue in the motif. In other cases, one basic residue was simply exchanged for another basic residue at position 10 or 24.

The final component of the network, comprising associations among the nodes N4, D28, and Q31, mapped to the base of the V3 loop. Although the network components {N5, T7, R30} and {N4, D28, Q31} are nested with respect to the amino acid sequence, preliminary analyses via molecular dynamics simulation of the V3 loop suggested that the side chains of D28 and Q31 occupied a distinct space apart from R30 (unpublished data). Thus, the consensus network components defined a stratified V3 loop with respect to its secondary structure, with clusters of putative interactions localized to its tip, stem, or base regions (Figure 4).

By mapping the statistical associations identified by edges in the consensus network to a structural model of the V3 loop 28, we were able to calculate the average distance separating the residue pairs with respect to the folded protein, or the number of residues separating the pair in the amino acid sequence (i.e., tertiary and primary distances, respectively). We also generated null distributions of mean distances by randomizing the residues occupying nodes of the consensus network. Although the observed mean primary distance (11 residues) coincided with the mean of the null distribution (10.8), the observed mean tertiary distance (10.1 Å) was significantly lower than expected (16.7 Å, *p* < 0.005; Figure S2). This discrepancy between the mean primary and tertiary distances indicated the influence of residue pairs bridging the V3 loop.

### Interactions between V3 and C4

**Figure 5:**
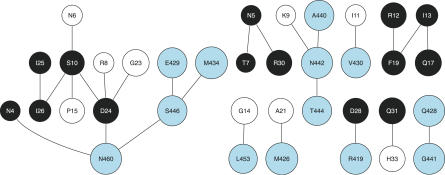
Combined V3 and C4 Network A consensus network assembled from edges with marginal posterior probabilities exceeding 0.95, in which nodes represent nonsynonymous substitution events at codon sites in the V3 and C4 domains. Nodes that correspond to residues in the C4 domain are shaded blue (numbered according to their position in the consensus *env* gene sequence), and nodes that were connected by strongly supported edges in the V3-specific network are shaded black. We retain the same numbering scheme for residues in V3 for consistency.

Residue–residue interactions between the V3 and C4 domains of gp120 have been documented in previous experimental work [69–72]. We reconstructed the evolutionary history of the C4 domain and merged the resulting matrix of substitution events with the matrix obtained from our analysis of V3 by rows (i.e., branches). A majority of the network components from our previous analysis of V3 were recovered in the consensus network of the merged V3–C4 dataset (Figure 5). (Residues in the C4 domain were numbered according to their location within the *env* gene of the HXB2 reference sequence. Because the HXB2 sequence contained several infrequent indels in the V3 domain, we retained our original numbering scheme of V3 residues for consistency.) Only the V3 network component {N4, D28, Q31} became disrupted by the inclusion of residues from the C4 domain. The residue–residue associations within V3 that this component comprised were replaced in the V3–C4 consensus network by a strongly supported association between D28 and the C4 residue R419. This shift also resulted in stronger evidence for a positive interaction between the V3 residues Q31 and H33. We found several strongly supported associations between the V3 and C4 domains of gp120, indicated by edges connecting residues located in either domain (e.g., D24–N460, G14–L453, and D28–R419; Figure 5). All edges in the V3–C4 consensus network represented positive associations. Mapping these residues to a three-dimensional model of the gp120 glycoprotein [28], we found that the D24 and N460 side chains were separated by a minimum distance of 3.6 Å (8.3 Å separating their α-carbons). Residue N442 was also located in close proximity to the V3 loop (to a minimum distance of 3.2 Å, or 4.0 Å between the α-carbons of N442 and N4), but remained a considerable distance from its putative interacting residue D24, suggesting that this putative interaction was more functional than structural in nature. Similarly, none of the other putative interactions between the V3 and C4 domains occurred between spatially clustered residues. We also detected a putative interaction between C4 residues that comprised a potential N-linked glycosylation site (N442–T444). This glycosylation motif was predominantly subtype C specific, but the majority of substitutions at these positions were mapped to branches outside the subtype C clade.

### Interactions Reflect Evolution within Hosts

Mapping substitutions within the V3 loop to branches in the tree allowed us to partition the analysis between terminal and internal branches, i.e., focusing on HIV-1 evolution within or among hosts, respectively. The network obtained from an analysis of substitutions mapped to terminal branches was very similar to the original network, recovering the edges N5–T7, S10–D24, R12–F19, and I13–Q17 (Figure S3). This result was consistent with a greater influence of the adaptation of HIV-1 within hosts on shaping sequence variation in V3. The network obtained from the map to internal branches displayed fewer similarities (Figure S3). We recovered the network component {N5, T7, R30} and the edge I25–I26 in our analysis of internal branches. We also found several edges that did not appear in the original network, e.g., I26–A32 and N5–P15. However, the network inferred from internal branches was sensitive to the low frequency of substitutions mapped to this portion of the tree (544 substitutions in total, compared to 3,751 substitutions mapped to terminal branches), and some edges may be spurious.

## Discussion

Our analysis of the covariation among residues comprising the V3 loop of the HIV-1 envelope glycoprotein gp120 is the first to model sequence variation as a joint probability distribution in a phylogenetic context. We refer to this type of analysis as an evolutionary-network model. By simulating sequences on the inferred phylogeny under a null model of independent evolution among sites, we show that analyses that do not account for common ancestry are susceptible to a high false-positive rate, even after applying corrections for multiple comparisons. Consequently, such analyses tend to over-report the number of significant associations within the V3 loop, ranging in one case from 39 to 157, depending on the association test statistic and method of adjusting for the false discovery rate [21]. This effect becomes even worse as one samples more sequences, which tends to increase the depth of the tree (i.e., time to the most recent common ancestor). Spurious associations may also result from indirect correlations between conditionally independent sites. For example, the V3 residues 10, 12, 19, 23, and 24 tend to be excessively interconnected by pairwise tests such that edges in the network form closed loops [19–21]. As a result, the networks assembled from all statistically significant pairwise associations tend to be over-connected and difficult to interpret.

Five out of the nine putative interactions that were identified by our evolutionary-network model have previously been reported in comparative studies. Pairing of residues 10 and 24 is ubiquitous [19–21], indicating that this interaction is sufficiently strong to overcome confounding effects of the phylogeny and statistical methodology. Residues 5 and 7 were also found to covary significantly by Bickel et al. [20], who also remarked on the presence of a glycosylation site. Finally, significant associations have been reported between the residue pairs 10–26 and 28–31 under some statistical tests conducted by Gilbert et al. [21]. These previous studies may have failed to detect the remaining putative interactions in our study because their analyses were susceptible to an elevated rate of false negatives, caused by raising the threshold of significance to control their inherently high false-positive rate.

Unfortunately, very few interactions between specific residues in the V3 loop have been described consistently by experimental or comparative studies (see Table S1). For example, de Jong et al. [15] found that substitutions at both site 10 and either 24 or 28 were necessary to restore the syncytium-inducing phenotype in chimeric HXB2 viruses propagated in T cell lines. Similarly, Shioda et al. [87] determined that substitutions at three to five positions in V3 (residues 12, 20, 21, 24, and 31) were required to modify the cell tropism phenotype of HIV-1; single amino acid substitutions were insufficient. Kuhmann et al. [73] used site-directed mutagenesis of an HIV-1 isolate to determine that substitutions at four different positions in V3 (residues 9, 12, 18, and 23) were necessary for the virus to become fully resistant to the CCR5-binding entry inhibitor AD101; however, it remains unclear whether the cumulative effect of these residues was nonadditive. The lack of concordance among experiments and comparative studies in identifying putative interactions between residues in V3 is due in part to assaying different phenotypes of V3 (e.g., cell tropism and coreceptor usage) whose genetic determinants may not overlap, as well as variation in the genetic and environmental context of V3 [74].

Similarly, there are few documented cases of interactions between specific residues in V3 and C4. Morrison et al. [70] found that a loss-of-function mutation (positionally equivalent to residue 434 in HIV-1) in the C4 domain of a simian immunodeficiency virus (SIVmac239) envelope glycoprotein gp120 could be compensated by a subsequent mutation in V3 (at residue 11). Subsequently, Kirchhoff et al. [71] identified two additional compensatory combinations between residues in V3 and C4 (including SIVmac239 *env* positions 334, 428, and 324). However, the HIV-1 and SIVmac239 *env* V3 loops are so divergent that comparisons are unlikely to be informative. In HIV-1, Carrillo and Ratner [72] found that substitutions at residues 430 and 441 in C4 restored the original cell-tropism phenotype in a V3-chimeric mutant.

In light of this, we have performed extensive tests to validate the accuracy of our model. Our simulations indicate that mapping substitution events to the phylogeny is very effective at removing the confounding influence of founder effects, reducing the high false-positive rate experienced by other methods by almost two orders of magnitude. In addition, complex patterns of conditional dependence among codon sites in V3 were revealed by our use of Bayesian network models. In sum, we find that the evolutionary-network model can reliably identify true interactions with a very low rate of false positives. Although our model is a considerable improvement over previous methods, it still requires a number of assumptions. First of all, we are mapping substitution events to branches in a tree that we assume to be a known quantity. It is possible to quantify this uncertainty by simultaneously sampling the topology of the tree and parameters of a nucleotide substitution model from a posterior probability distribution [75,76]. But sampling the parameters of a codon substitution model for a very large tree (over a thousand leaf nodes) is an extremely computationally demanding task and may be exceedingly slow to converge. Furthermore, previous studies have demonstrated that maximum-likelihood estimates of substitution rates or counts are relatively insensitive to the tree topology [51,77].

Secondly, we implicitly assume that our codon substitution model is an accurate representation of the true process underlying the evolution of our sample of HIV-1 *env* sequences. Like the vast majority of evolutionary models, this particular model assumes that the evolutionary process at one codon position is independent of all others. In other words, we are using a model that assumes the absence of interactions among sites in order to map substitutions to branches, which in turn will be used to detect interactions among sites. One could argue that this assumption may limit the sensitivity of our study to detect interactions. However, previous work suggests that the accuracy in mapping substitutions to branches in the tree is robust to the failure to account for such interactions. An interaction between sites will be manifested as variation in substitution rates over time, also known as “heterotachy” [78]. Phylogenetic methods based on ancestral reconstruction have been demonstrated to be robust to heterotachy [37] and variation in substitution rates across sites as well [51]. In the absence of prior knowledge, the independent-sites model is more conservative because it is not biased toward identifying particular interactions among sites.

Third, our application of the evolutionary-network model to V3 loop sequences implicitly assumes that residue–residue interactions are constant throughout the evolutionary history of the sequences. This assumption is susceptible to subtype-specific interactions [21] including other domains of the gp120 glycoprotein [69,71,72,79,80], which may become masked by pooling data from multiple contexts. For example, substitutions at some sites involved in interactions identified in this study (i.e., 5 ↔ 30, 25 ↔ 26, and 4 ↔ 31) tend to map to branches in the subtype D clade. Upon inspection, however, none of the statistical associations in the model appear to be exclusive to any one subtype (unpublished data). In fact, our model does not preclude the analysis of full-length *env* sequences, nor the inclusion of other factors, which would simply introduce additional variables into the Bayesian network. Such an analysis would address the possibility of subtype-specific interactions. Despite our implementation of a network-learning procedure that is tailored to handle limited datasets [52], however, our sample of sequences (*n* = 1,154) is barely sufficient to support such an analysis, which would potentially involve up to 721 codon positions (omitting variable loops V1/V2, V4, and V5). Instead, we have intentionally restricted our main analysis to residues within the V3 loop in order to contrast our methods against those of previous comparative studies analyzing the covariation within this domain [19–21]. Moreover, the edges of the V3-specific network are largely robust to the inclusion of the C4 domain into our analysis, suggesting that these putative interactions in V3 are mostly independent of subtype variation in the remainder of the *env* gene.

Finally, our analysis of covariation in V3 handles all nonsynonymous substitutions at a given site equivocally, i.e., making no distinction between the specific residues involved. This approximation greatly reduces the dimensionality of the model to binary states (presence or absence of any nonsynonymous substitution). As in the case of subtype-specific interactions, this approximation could potentially mask residue-specific interactions [64]. However, the presence of residue-specific interactions does not necessarily prevent our analysis from detecting a statistical association between the sites overall. For example, our model identifies a putative interaction between residues 5 and 7 in V3, even though the reconstructed substitutions specifically target residues defining an N-linked glycosylation motif.

The paradigm of a subdivision of function among sections of the V3 loop originated with experiments identifying the “tip” region as the principal neutralizing determinant [3]. Sequence variability localized to the strands immediately flanking the tip further suggested that the V3 loop could be partitioned into three functional regions: a conserved tip and base, and a flexible stem [28,81]. Subsequent experimental work has implicated residues in the conserved base of the V3 loop (residues 2–7 and 25–30) in specific interactions with the sulfated tyrosines of the N-terminal region of the CCR5 coreceptor [82], spatially distinct from the region bound by the tip [83]. Our comparative study of covariation in V3 sequences corroborates this empirically motivated model of a functionally stratified V3 loop. (However, we note that although comparative studies of sequence covariation can detect interactions between residues, they cannot distinguish functional from structural interactions.)

Ultimately, our goal is to map residue–residue interactions to host factors and clinically relevant virus phenotypes, such as coreceptor usage or neutralization sensitivity. Because our unit of observation consists of inferred evolutionary events rather than observed variation, we will require an evolutionary model for every phenotype to be included in the analysis, including continuous traits. The task of detecting interactions among components of genotype or phenotype has rapidly grown in its significance to HIV-1 research. Outside of the ongoing work on associating sequence variation in the V3 loop with coreceptor usage [16,35], investigators have applied several types of association tests to study covariation within the HIV-1 protease [62] and reverse transcriptase in association with drug resistance [61,84,85], and the Gag polyprotein [86]. None of these studies place interactions in an evolutionary context, and have only recently been able to address higher-order interactions. In light of our analysis of the V3 loop, we believe that application of the evolutionary-network model will provide new insight into diverse aspects of HIV-1 biology.

## Supporting Information

Figure S1 Consensus Bayesian Network for HIV-1 Protease This network was assembled from edges with marginal posterior probabilities exceeding a cutoff of 0.95, obtained from applying our evolutionary-network method to an alignment of HIV-1 subtype B protease sequences. Edges are labeled with their marginal posterior probability expressed as a percentage. Nodes are labeled with the alignment consensus residue and position of the codon site (consistent with the HXB2 reference sequence). Codon sites that have been previously implicated in resistance to protease inhibitors or subsequent compensatory mutations are labeled in pink (cross-resistant) or orange (specific to nelfinavir).
(10 KB PDF)

Figure S2 Null Distribution of Tertiary and Primary Distances This contour plot was generated from random assignments of V3 residues to nodes of the consensus network. Mean primary distance (*x*-axis) was calculated from the number of residues separating the pairs of residues connected by an edge in the network in the amino acid sequence. Mean tertiary distance (*y*-axis) was calculated from the structural coordinates of residues in the PDB model 2B4C [28]. The observed means are indicated on the plot as an open circle.
(15 KB PDF)

Figure S3 Terminal and Internal Branch-Specific Networks (A) A maximum-likelihood network obtained from substitutions mapped to terminal branches of the tree. Each node corresponds to a residue in the V3 domain, numbered according to its position in the consensus sequence and labeled with the consensus amino acid. Edges connecting nodes indicate an interaction between residues.
(B) A consensus network obtained from substitutions mapped to internal branches of the tree. Edges are labeled with their corresponding parametric bootstrap support values.
(189 KB PDF)

Table S1 Contrasting Experimental and Comparative Studies of Interactions in V3 In this table, we summarize the evidence for various putative interactions between pairs of residues in the HIV-1 envelope V3 loop. C = evidence from comparative studies of V3 sequences, E = evidence from experimental mutagenesis of V3 sequences. Entries in parentheses indicate putative interactions within intervals of the V3 sequence that were not completely resolved to specific residue pairs. Overall, concordance among studies is poor, with the possible exception of associations between the residues S10, R12, and D24. Citations for each study were abbreviated as follows: K93 = Korber et al. (1993) [19]; B96 = Bickel et al. (1996) [20]; G05 = Gilbert et al. (2005) [21]; deJ92 = deJong et al. (1992) [15]; S92 = Shioda et al. (1992) [87]; W92 = Westervelt et al. (1992) [88]; F92 = Fouchier et al. (1992) [44]; C92 = Chesebro et al. (1992) [89]; and C96 = Chesebro et al. (1996) [74]. We note that concordance between Korber et al. (1993) and Bickel et al. (1996) may be explained by significant overlap in sequence data and methodological criteria.
(52 KB PDF)

### Accession Number

The Research Collaboratory for Structural Bioinformatics Protein Data Bank (http://www.rcsb.org/) accession number for the V3 loop of HIV-1 gp120 complexed to the CD4 receptor and X5 antibody is 2B4C.

## Abbreviations

BDe: Bayesian Dirichlet metric
HIV-1: human immunodeficiency virus type 1
V3: third variable domain
