# Supplementary material for: An Evolutionary-Network Model Reveals Stratified Interactions in the V3 Loop of the HIV-1 Envelope
Source: PLoS Comput Biol. 2007 Nov 23;3(11):e231. doi: 10.1371/journal.pcbi.0030231 (PMC2082504; doi:10.1371/journal.pcbi.0030231)
Supplement: Supplementary file 1 [file pcbi.0030231.sg001.pdf]

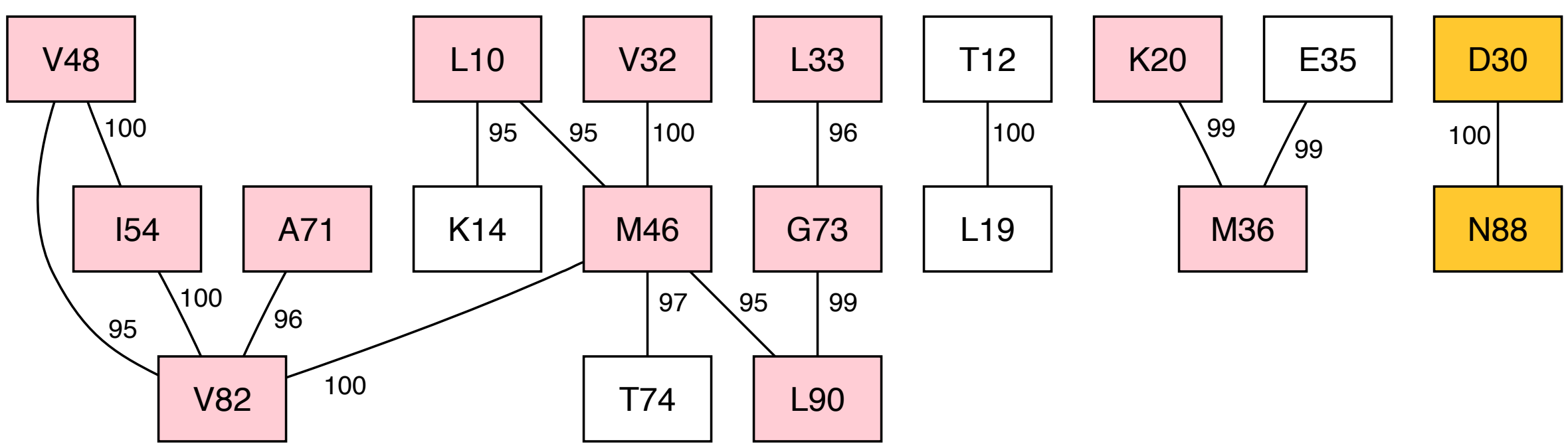
V48
L10
V32
L33
T12
K20
E35
D30
I54
A71
K14
M46
G73
L19
M36
N88
V82
T74
L90
100
95
95
100
96
100
99
99
100
100
96
95
100
97
95
99
