# Supplementary material for: An Evolutionary-Network Model Reveals Stratified Interactions in the V3 Loop of the HIV-1 Envelope
Source: PLoS Comput Biol. 2007 Nov 23;3(11):e231. doi: 10.1371/journal.pcbi.0030231 (PMC2082504; doi:10.1371/journal.pcbi.0030231)
Supplement: Supplementary file 2 [file pcbi.0030231.sg002.pdf]

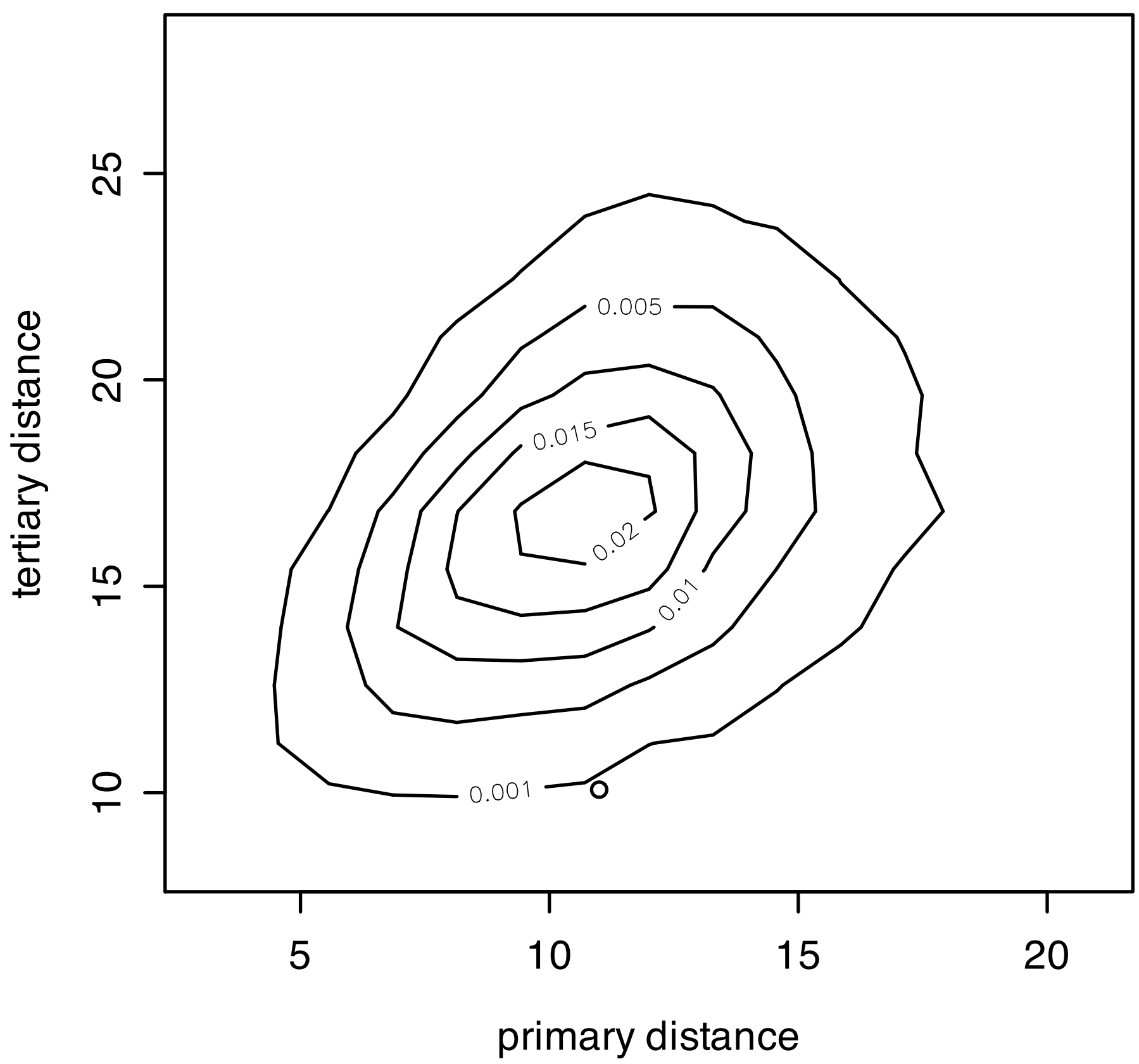

0.005
0.015
0.02
0.01
0.001
tertiary distance
25
20
15
10
5
10
15
20
primary distance
