# Supplementary material for: An Evolutionary-Network Model Reveals Stratified Interactions in the V3 Loop of the HIV-1 Envelope
Source: PLoS Comput Biol. 2007 Nov 23;3(11):e231. doi: 10.1371/journal.pcbi.0030231 (PMC2082504; doi:10.1371/journal.pcbi.0030231)
Supplement: Supplementary file 3 [file pcbi.0030231.sg003.pdf]

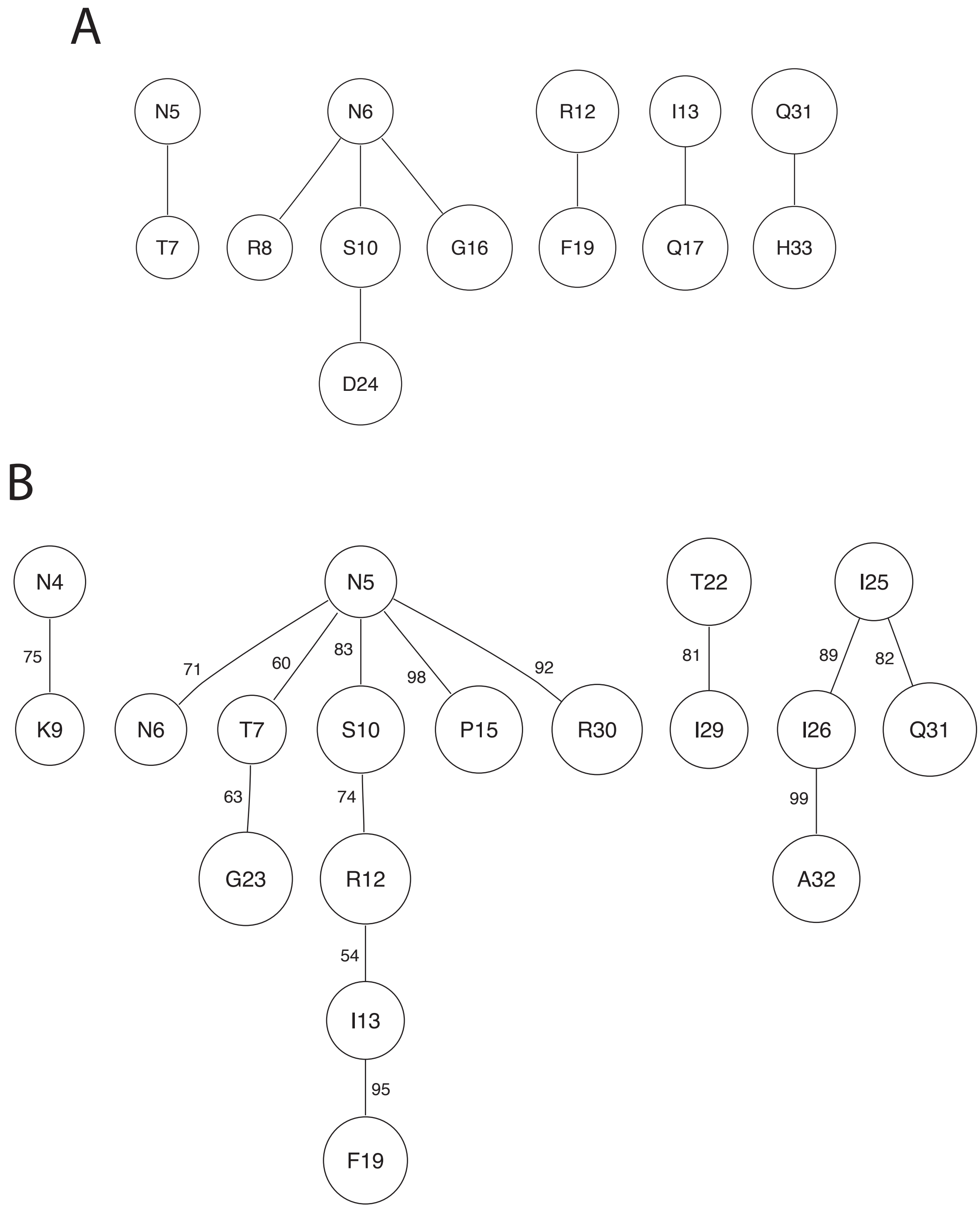
A
N5
T7
N6
R8
S10
G16
D24
R12
F19
I13
Q17
Q31
H33
B
N4
75
K9
N5
71
60
83
98
92
N6
T7
S10
P15
R30
63
74
G23
R12
54
I13
95
F19
T22
81
I29
I25
89
82
I26
Q31
99
A32
