# Supplementary material for: An Evolutionary-Network Model Reveals Stratified Interactions in the V3 Loop of the HIV-1 Envelope
Source: PLoS Comput Biol. 2007 Nov 23;3(11):e231. doi: 10.1371/journal.pcbi.0030231 (PMC2082504; doi:10.1371/journal.pcbi.0030231)
Supplement: Supplementary file 4 [file pcbi.0030231.st001.pdf]

| | | This study | K93 | B96 | G05 | deJ92 | S92 | W92 | F92 | C92 | C96 |
|---|---|---|---|---|---|---|---|---|---|---|---|
| T1 | G23 | | | | C | | | | | | |
| N4 | T7 | | | C | | | | | | | |
| N4 | Y20 | | | | C | | | | | | |
| N4 | Q31 | C | | | | | | | | | |
| N5 | T7 | C | | C | | | | | | | |
| N5 | K9 | | | C | | | | | | | |
| N5 | I26 | | | C | | | | | | | |
| N5 | D28 | | | C | | | | | | | |
| N5 | R30 | C | | | | | | | | | |
| N6 | I26 | | | C | | | | | | | |
| T7 | K9 | | | C | | | | | | | |
| T7 | S10 | | | C | | | | | | | |
| T7 | I26 | | | C | | | | | | | |
| T7 | D28 | | | | C | | | | | | |
| T7 | A32 | | | | C | | | | | | |
| R8 | S10 | | | C | C | | | | | | |
| R8 | D24 | | | | C | | | | | | |
| S10 | R12 | | C | C | | | | | | | |
| S10 | F19 | | | C | | | | | | | (E) |
| S10 | G23 | | | | C | | | | | | |
| S10 | D24 | C | C | C | C | E | | | E | | |
| S10 | I26 | C | | | | | | | | | |
| S10 | G27 | | | | C | | | | | | |
| S10 | D28 | | | | | E | | | | | |
| I11 | P15 | | | C | | | | | | | |
| I11 | T18 | | | C | | | | | | | |
| R12 | T18 | | C | C | | | | | | | |
| R12 | F19 | C | | C | | | | | | | (E) |
| R12 | G23 | | C | | | | | | | (E) | |
| R12 | D24 | | C | C | | | (E) | E | | (E) | (E) |
| I13 | Q17 | C | | | | | | | | | |
| P15 | F19 | | | | C | | | | | | |
| T18 | F19 | | | | C | | | | | | |
| F19 | D24 | | C | C | | | | | | | |
| Y20 | A21 | | | | C | | (E) | | | | |
| Y20 | D24 | | | | C | | (E) | | | | |
| G23 | D24 | | C | | C | | | | | | |

| | | | | |
|---|---|---|---|---|
| D24 | G27 | | | C |
| I25 | I26 | C | | |
| D28 | R30 | | C | |
| D28 | Q31 | C | | |

**Table S1.**
